# The Situation: A Paradox

**Published:** 1903-11

**Authors:** 


### TEXAS MEDICAL JOURNAL.

AUSTIN, TEXAS.

A MONTHLY JOURNAL OF MEDICINE AND SURGERY.

EDITED AND PUBLISHED BY
F. E. DANIEL, M. D.

ASSOCIATE EDITORS:

WITTEN BOOTH RUSS, M. D., San Antonio, Texas.

P. M. PAYNE, M. D., Brownwood, Texas.

Published Monthly at Austin, Texas. Subscription price $1.00 a year in advance.

Eastern Representative: John Guy Monihan, St. Paul Building, 220 Broadway, New York City.

Official organ of the the West Texas Medical Association, the Houston District Medical Association, the Austin District Medical Society, the Brazos Valley Medical Association, the Galveston County Medical Society, and several others.

### THE SITUATION: A PARADOX.

On the 12th of Ooctober there had been at Laredo, as per official report, 210 cases of yellow fever, and 10 deaths—5 per cent. On the 12th of November there had been 789 cases and 78 deaths—10 per cent. True to the history of the disease, it has "grown with what it feeds upon," and has raged like a fire, and is, at this writing, still raging, and it will continue to rage so long as there is material to feed it. In the first thirty days there occurred 211 cases. In the next thirty days there occurred 579 cases. The ratio of deaths to this accession was (579 cases, 68 additional deaths) 12 per cent. At this writing (November 12th) new cases are occurring at the rate of twenty to twenty-five a day. Every house in Laredo has been fumigated. I infer that the fumigation has included the use of pyrethrum, the best mosquitocide. The town has been cleaned up; screens and bars have been "used for all they are worth"; oil has been repeatedly put on over 2000 barrels and cisterns of water, and all stagnant water had been drained. United States government and State experts have been constantly on the ground, and every resource known to science has been brought into action to arrest the spread of the disease, all in vain.

If the mosquito is the sole means of spreading the fever, why has the fever steadily and rapidly spread despite these efforts?

Meantime the infection got out of Laredo, somehow. Five cases occurred near Cuero, three fatal. Two hundred occurred at Minerva, with a pretty heavy mortality.

It was introduced into San Antonio, so it is said, under conditions pre-eminently favorable to its spread—that is, under similar and even worse conditions than existed at Laredo.

The history of every epidemic of yellow fever of which I have any knowledge shows that when the poison is introduced into a locality where there are a large number of non-immunes, in a warm climate, in hot weather, an epidemic occurs, and it has never been known to be (I had nearly said "stamped out") arrested. It was introduced into San Antonio under those circumstances. There were many thousand visitors in the town, all presumably susceptible. It is a semi-tropical climate. The weather was hot and remained so, and still is far from cool; the sanitary condition was bad; a large contingent of the resident population are, as in Laredo, very poor, dirty Mexicans, who live on scant and unwholesome rations, and the best possible breeding ground for mosquitoes is afforded by the dirty little serpentine stream that winds around and around through the town, stagnant in many places in summer. In fact, there was every reason to believe and fear that in thirty days from the occurence of the first case there would be an epidemic of fearful proportions raging there, and as the visitors scattered all over Texas at the first alarm there was serious reason to fear a repetition of the death-dealing widely-scattered epidemic of 1867, which invaded most of the Texas railroad towns south of 32 degrees latitude. Fortunately these anticipations were not realized, and the escape, in my opinion, is as remarkable as it is happy. The fever has gone back on its record. It did not become epidemic in San Antonio. Why?

The experience at Laredo, it seems to me, has demonstrated the incorrectness of the claim that the mosquito is the only factor in spreading the disease. Had it been, it would (or rather should) never have become such a raging epidemic. The authorities should have been able, with the measures used, to restrain it; and while I have not seen any of the cases diagnosed as yellow fever at San Antonio, and am not in position to give an opinion on that point, and can not say as did the old preacher, "I beg to differ with Brother Paul," reasoning upon the history of epidemics, with which I am personally somewhat familiar by experience, I will say the fact that the disease did not spread furnishes presumptive evidence that there might have been a mistake in the diagnosis. The physicians at San Antonio are not by any means a unit on the subject.

If the mosquito is the sole agent in yellow fever epidemics, why did they not spread it in San Antonio? They were not as effectually dealt with as in Laredo.

